# The Bi-Functional Paxilline Enriched in Skin Secretion of Tree Frogs (*Hyla japonica*) Targets the KCNK18 and BK_Ca_ Channels

**DOI:** 10.3390/toxins15010070

**Published:** 2023-01-12

**Authors:** Chuanling Yin, Fanpeng Zeng, Puyi Huang, Zhengqi Shi, Qianyi Yang, Zhenduo Pei, Xin Wang, Longhui Chai, Shipei Zhang, Shilong Yang, Wenqi Dong, Xiancui Lu, Yunfei Wang

**Affiliations:** College of Wildlife and Protected Area, Northeast Forestry University, Harbin 150040, China

**Keywords:** tree frogs, skin secretion, paxilline, KCNK18, defensive strategy

## Abstract

The skin secretion of tree frogs contains a vast array of bioactive chemicals for repelling predators, but their structural and functional diversity is not fully understood. Paxilline (PAX), a compound synthesized by *Penicillium paxilli,* has been known as a specific antagonist of large conductance Ca^2+^-activated K^+^ Channels (BK_Ca_). Here, we report the presence of PAX in the secretions of tree frogs (*Hyla japonica*) and that this compound has a novel function of inhibiting the potassium channel subfamily K member 18 (KCNK18) channels of their predators. The PAX-induced KCNK18 inhibition is sufficient to evoke Ca^2+^ influx in charybdotoxin-insensitive DRG neurons of rats. By forming π-π stacking interactions, four phenylalanines located in the central pore of KCNK18 stabilize PAX to block the ion permeation. For PAX-mediated toxicity, our results from animal assays suggest that the inhibition of KCNK18 likely acts synergistically with that of BK_Ca_ to elicit tingling and buzzing sensations in predators or competitors. These results not only show the molecular mechanism of PAX-KCNK18 interaction, but also provide insights into the defensive effects of the enriched PAX.

## 1. Introduction

The skin of frogs is crucial to the survival and adaptability of these amphibians to various habitats and ecological conditions. Besides respiration, water regulation and antimicrobial and antifungal resistance, the skin of frogs serves many other functions. For example, several bioactive molecules have been identified from the secretion for deterring predators, such as toxic alkaloids [1,2,3,4] and peptide toxins [5,6,7]. Except for gene-encoding peptides, most chemical compounds are not directly synthesized by frogs. Alternatively, the frogs can either sequester these compounds unchanged from dietary sources into their skin glands or equip microbes with the necessary machinery to produce them [8,9]. Therefore, natural communities of microbes and fungi, such as those that live on amphibians’ skin, are essential for enhancing chemical defenses [10]. Fungi produce various secondary metabolites with unique and complex structures. Among them, indolo-terpenoids of the paxilline (PAX) type belong to a large family of secondary metabolites that exhibit unique molecular architectures and diverse biological activities [11]. PAX has been known as a potent blocker of BK_Ca_ (large-conductance voltage- and Ca^2+^-activated K^+^) channels, generally acting at low nanomolar concentrations [12]. Accordingly, the application of PAX induced tremors in rodents was sustained for several hours [13], suggesting the potent toxicity of this molecule.

Although with excellent jumping ability, some tree frogs with small body sizes are considered vulnerable prey in the arboreal habitat. In this case, several gene-encoded toxins have evolved to serve as chemical weapons to deter potential terrestrial predators [14]. Unlike the peptide toxins identified from typical venomous animals [15,16,17,18], most amphibia-derived peptides show less potency to their target [19]. Is there symbiotic fungus on the skin of tree frogs to facilitate this defensive strategy? In this study, we identified PAX from the skin secretion of tree frogs, suggesting the existence of symbiotic fungi that produce toxic chemicals. Furthermore, our functional tests demonstrated that KCNK18 acts as a novel target of PAX. By inhibiting both BK_Ca_ and KCNK18 channels of predators, PAX exhibits critical biological significance to evoke excitatory currents on sensory neurons and elicit tingling and buzzing sensations in predators or competitors. Therefore, our results show the existence of bi-functional PAX in the frog skin secretion, which may represent a unique defense mechanism in amphibian adaptation.

## 2. Results

### 2.1. PAX Evokes Ca^2+^ Signals on ChTx-Insensitive Neurons

**Figure 1:**
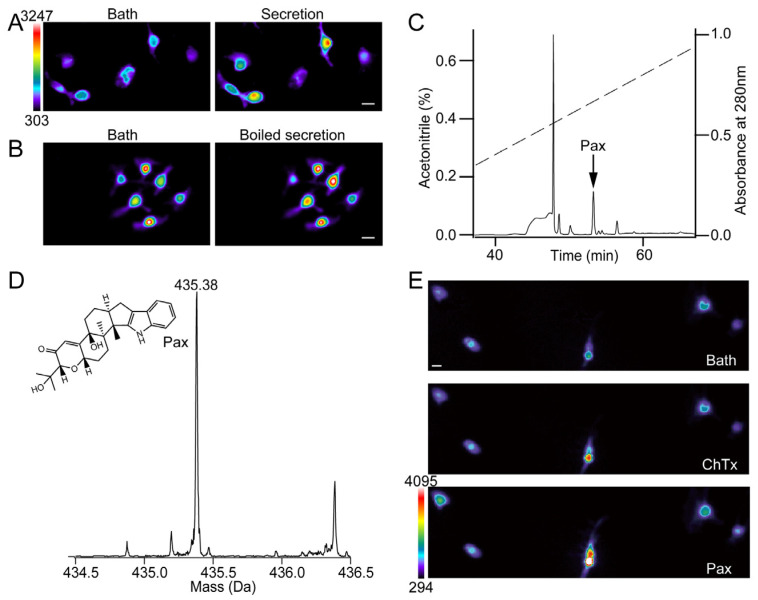
PAX induces Ca^2+^ signals on ChTx-insensitive neurons. (**A**,**B**) Representative calcium increase of rat DRG neurons in the presence of intact (**A**) or boiled (**B**) skin secretions of tree frogs. Scale bar, 50 μm (horizontal), 303 to 3247 AU (vertical). (**C**) Isolation of native PAX (black arrow) from frog skin secretions by a C_18_ RP-HPLC column. (**D**) The chemical structure and molecular weight of PAX. (**E**) Calcium imaging of rat DRG neurons in the presence of 100 nM ChTx and 20 μM PAX. Scale bar, 50 μm (horizontal), 294 to 4095 AU (vertical).

By using calcium imaging, we assessed the effect that the collected secretions of tree frogs (*Hyla japonica*) would have on the dorsal root ganglion (DRG) of rats (*Rattus norvegicus*). Interestingly, we found that more than half of these neurons exhibited robust calcium signals in the presence of 20 mg/mL secretion (Figure 1A). To rule out the possibility that pore forming peptides may disrupt the membrane architecture to elicit the calcium influx [20], we used boiled secretion and again carried out the calcium imaging test. Similarly, the boiled secretion showed equal efficacy in evoking calcium signals (Figure 1B), suggesting that chemical compounds are likely to be responsible for this activity. We therefore employed high performance liquid chromatography (HPLC) to purify the active compound (Figure 1C). The results from LC-MS (Liquid Chromatography-Mass Spectrometry) and functional tracking indicated that PAX molecules bestowed the secretion with activity for eliciting calcium influx on neurons (Figure 1D). Although PAX and charybdotoxin (ChTx) have been known as the blockers of BK_Ca_ channels, we found that PAX is sufficient to evoke Ca^2+^ influx in ChTx-insensitive neurons (Figure 1E). These results suggest that PAX can target other receptors, except BK_Ca_ channels.

### 2.2. PAX Exhibits an Inhibitory Effect on Rat KCNK18

**Figure 2:**
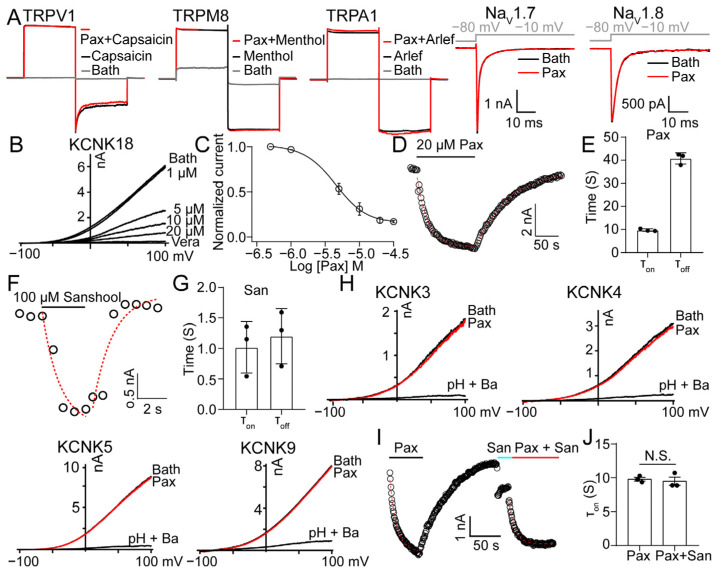
PAX selectively inhibits rat KCNK18. (**A**) 100 μM PAX had no inhibitory effect on TRPV1, TRPM8, TRPA1, Na_V_1.7 and Na_V_1.8. (**B**) Representative KCNK18 currents inhibited by PAX at different concentrations. 100 μM verapamil (vera) was used as a positive control. (**C**) Dose–response relationship of PAX inhibiting KCNK18. Data were fitted to a Hill equation (average ± SEM; *n* = 3 for each data point). (**D**) Representative wash-in and wash-out time course of 20 μM PAX on KCNK18 recorded at 100 mV, superimposed with fittings of a single-exponential function (red dotted curves). (**E**) The associated and dissociated time of 20 μM PAX on KCNK18 (average ± SEM; *n* = 3). (**F**) Representative wash-in and wash-out time course of 100 μM sanshool on KCNK18 recorded at 100 mV, superimposed with fittings of a single-exponential function (red dotted curves). (**G**) The associated and dissociated time of 100 μM sanshool on KCNK18 (average ± SEM; *n* = 3). (**H**) 100 μM PAX had no inhibitory effect on KCNK3, KCNK4, KCNK5 and KCNK9. (**I**) Representative wash-in time course of 20 μM PAX on KCNK18 recorded at 100 mV in the absence and presence of 50 μM sanshool, superimposed with fittings of a single-exponential function (red dotted curves). The solid black line indicates the presence of 20 μM PAX. The solid blue line indicates the presence of 50 μM sanshool. (**J**) The associated time of 20 μM PAX on KCNK18 in the absence and presence of 50 μM sanshool (average ± SEM; *n* = 3; N.S., no significance).

By using electrophysiological recordings, we screened several ion channels highly expressed in rat DRG neurons (Figure 2A). Among them, KCNK18 was potently inhibited by PAX (Figure 2B,C), yielding an IC_50_ value of 4.13 ± 0.04 μM. A kinetic analysis of PAX-induced KCNK18 inhibition revealed that the high affinity was due to a combination of rapid binding and very slow unbinding (Figure 2D,E). The washing out time course (τ_off_) of PAX recorded from whole-cell configuration is 30.6 ± 2.1 s, which is much longer than that of sanshool (Figure 2F,G), a well-known KCNK18 inhibitor isolated from Szechuan peppercorns [21]. Furthermore, 100 μM PAX showed no effect on KCNK3, KCNK4, KCNK5 and KCNK9 (Figure 2H), demonstrating that PAX exhibits subtype selectivity among KCNK channels. We next used competing assays to investigate the interaction between PAX and KCNK18 to test whether PAX shares a similar binding pocket with sanshool. As illustrated in Figure 2I,J, the rate of PAX association was 11.4 ± 0.23 s, which was relatively slower than that of sanshool (τ_on_ = 1.15 ± 0.12 s). In addition, 20 μM PAX produced a blockage of KCNK18 currents after pretreatment with 50 μM sanshool (Figure 2I). However, the rate of blockage by PAX was intact (Figure 2J). This raised the possibility that PAX does not interact with the binding sites of sanshool, although none have yet been elucidated.

### 2.3. Key Residues for PAX-KCNK18 Interaction

**Figure 3:**
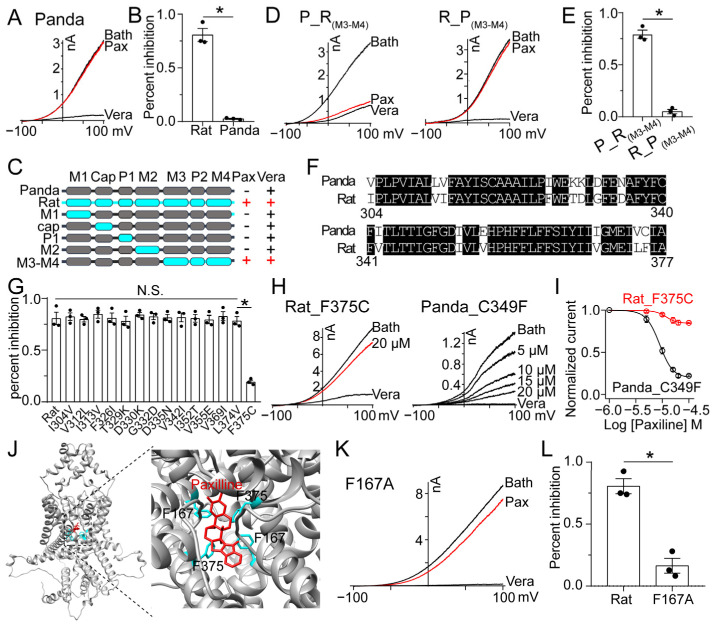
The binding pocket of PAX to KCNK18. (**A**) 20 μM PAX had no inhibitory effect on the panda KCNK18 channel. (**B**) The inhibitory rate of 20 μM PAX on rat and panda KCNK18 (average ± SEM; *n* = 3; * *p* < 0.01). (**C**) Schematic representation of the chimeras between panda (grey) and rat (cyan) KCNK18. The responses of wild-type and chimeric KCNK18 channels to 20 μM PAX and 100 μM vera are given. (**D**) Representative currents of chimeric KCNK18 inhibited by 20 μM PAX and 100 μM vera. P_R(M3-M4) represents that the M3-M4 region of panda KCNK18, was replaced by the homologous region of rat KCNK18, and vice versa R_P(M3-M4). (**E**) The inhibitory rate of 20 μM PAX on chimeric KCNK18 channels (average ± SEM; *n* = 3; * *p* < 0.01). (**F**) The sequence alignment of M3-M4 domain of panda and rat KCNK18. (**G**) The inhibitory rate of 20 μM PAX on rat KCNK18 mutants (average ± SEM; *n* = 3; N.S., no significance; * *p* < 0.01). (**H**) Representative currents of KCNK18 mutants inhibited by PAX and 100 μM vera. (**I**) Dose–response relationship of PAX inhibiting KCNK18 mutants. Data were fitted to a Hill equation (average ± SEM; *n* = 3). (**J**) The side view of PAX docking to rat KCNK18 model (left). The binding pocket of PAX was enlarged (right), and four phenylalanine are located near PAX. (**K**) Representative currents of rat KCNK18 mutants inhibited by 20 μM PAX and 100 μM vera. (**L**) The inhibitory rate of 20 μM PAX on rat KCNK18 and F167A mutant (average ± SEM; *n* = 3; * *p* < 0.01).

To study the interaction between PAX and KCNK18, we screened the PAX effect on KCNK18 orthologs. Interestingly, panda KCNK18 was insensitive in the presence of PAX (Figure 3A,B). By using the chimeras constructed between rat and panda KCNK18 channels (Figure 3C), we found that the rat M3-M4 segment bestowed panda KCNK18 with PAX sensitivity, while rat KCNK18 containing panda M3-M4 segment had no response to PAX (Figure 3D,E). Therefore, the segment from M3 to M4 served as a transplantable domain to exhibit PAX sensitivity. Among the segment, only 14 residues differ between rat and panda KCNK18 channels (Figure 3F). Importantly, site 375 (in rat KCNK18 channel) is likely responsible for the interaction between KCNK18 and PAX, given that swapping this homologous site could either abolish or establish the PAX sensitivity on rat and panda KCNK18, respectively (Figure 3G–I). We therefore constructed the structural model of rat KCNK18 and tested residues nearby site 375 to screen other key amino acids that may also participate in the interaction between KCNK18 and PAX (Figure 3J). As shown in Figure 3K,L, the point mutant F167A lost the PAX sensitivity, suggesting that the side chains of F167 and F375 are likely to interact with PAX. Therefore, the interaction between PAX and KCNK18 is largely due to the π-π stacking interactions, which lead to the PAX blockage in the ion permeation pathway.

### 2.4. The Conserved Binding Pocket for PAX Binding

**Figure 4:**
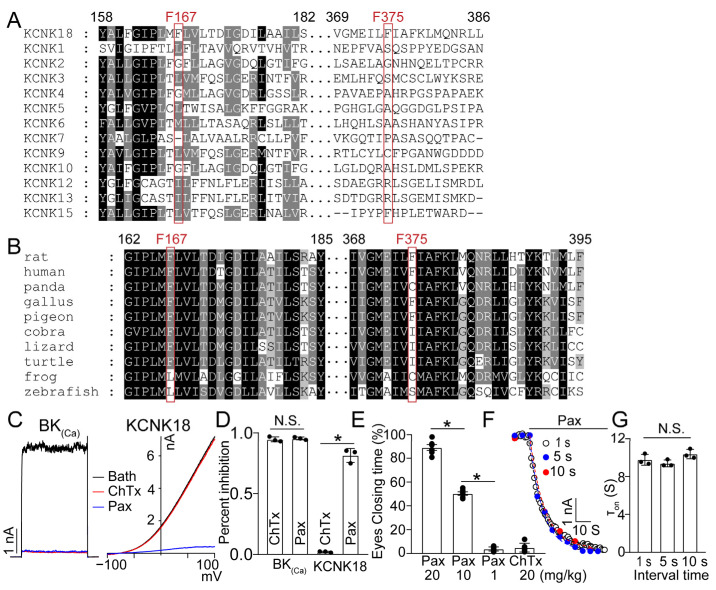
The sequential and functional comparison of KCNK families. (**A**) The sequence alignment of rat KCNK families. (**B**) The sequence alignment of KCNK18 orthologs. (**C**) Representative currents of BK_(Ca)_ and KCNK18 inhibited by 1 μM ChTx (red) and 20 μM Pax (blue). (**D**) The inhibitory rate of 1 μM ChTx and 10 μM Pax on BK_(Ca)_ and KCNK18 (average ± SEM; *n* = 3; * *p* < 0.01). (**E**) Eye closing response of rat after intravenous tail injection of PAX or ChTx. The time of both eyes closing was calculated (average ± SEM; *n* = 6; * *p* < 0.01). (**F**) Representative wash-in time course of 20 μM PAX on rat KCNK18. The perfusion of 20 μM PAX was constantly applied (indicated by a black bar). For evaluation of the inhibitory effect of PAX, the current was evoked from the holding potential (−80 mV) by the test pulse at +100 mV. The interval between each sweep was 1, 5 or 10 s, respectively. The currents were superimposed with fittings of a single-exponential function. (**G**) The associated time of 20 μM PAX on KCNK18 at different intervals of stimuli (average ± SEM; *n* = 3; N.S., no significance).

Among the KCNK family, none of the other subtypes possesses conserved phenylalanine at two key positions (Figure 4A), which may bestow PAX with subtype-selectivity. On the contrary, both phenylalanines are highly conserved in most of the avian and mammalian KCNK18 orthologs (Figure 4B), implying that the PAX equipped by tree frogs exerts general bioactivity against KCNK18 of potential predators or encounters. To evaluate the in vivo effect of the bi-functional PAX, we used ChTx, a BK_Ca_ inhibitor without targeting KCNK18 (Figure 4C,D), as the control. To measure the pain reactions induced by KCNK dysfunction, the eye closure was monitored to assess the painful sensation of rat evoked by ChTx or PAX [22]. Compared with ChTx, the application of PAX elicited much more painful responses in the rat model (Figure 4E). Therefore, our results suggest that the inhibition of KCNK18 induced by PAX likely acts synergistically with its suppression of BK_Ca_ to elicit tingling and buzzing sensations.

## 3. Discussion

Animals maintain a symbiotic relationship with commensal microbes, which allows them to regulate their biological operations by using functional molecules from these microbes. For instance, several marine protostomians and deuterostomians use symbionts to produce tetrodotoxin (TTX) from microbes [23]. Due to the absence of enzymes responsible for PAX biosynthesis, it is also likely that the PAX found in the skin secretions of tree frogs originates from symbiotic microbes, especially fungi. As a tremorgenic mycotoxin, PAX shows a robust inhibitory effect on BK_Ca_ channels [12]. In this work, our results demonstrate a novel target of PAX, which may promote the toxic effect induced by the blockage of BK_Ca_ channels. PAX-induced inhibition of KCNK18 currents persisted for minutes of washout (Figure 2D,E), which causes prolonged K^+^ ions’ retention in the cytoplasm of sensory neurons and continuous cell excitation [24]. The physiological effect of KCNK18 inhibition can be referred to as sanshool, the active compound from Szechuan peppers eliciting a unique sensation that is best described as tingling paresthesia or numbing [25]. It has been known that downregulated *kcnk18* mRNA or KCNK18 loss of function plays a crucial role in neuropathic pain [26], suggesting that the PAX-induced KCNK18 inhibition could evoke painful sensation in predators of the frogs. Due to the bifunction that targets BK_Ca_ and KCNK18 channels, we assumed that the defensive effect of PAX produced by symbiotic fungi was underestimated in previous reports.

The binding pocket of PAX on KCNK18 locates in the cavity region of the ion permeation pathway, which is likely responsible for the very slow dissociation (Figure 3J). Furthermore, our results emphasize the role of phenylalanines in PAX-KCNK18 interaction, given that mutation on either F167 or F375 (in rat KCNK18 channel) disrupted the inhibitory effect of PAX. In agreement with this, the binding models of other chemicals containing benzene rings have suggested a similar binding pocket [27], although the affinity of these chemicals is much lower than that of PAX. Interestingly, PAX did not respond to the state of KCNK18 (Figure 4F,G) in order to access this pocket, implying that the entry of PAX was directed from the bottom of this channel. According to the different binding mechanisms of PAX on KCNK18 and BK_Ca_ channels, PAX can be used as a template to develop selective KCNK18 inhibitors.

It is fascinating how animals are widely exposed to exogenous chemicals. In this case, the employment of PAX-producing microbes by tree frogs is expected to boost the toxicity of their skin secretion. One or a few transport proteins (toxin sponge molecules) in poison frogs acquired the ability to sequester toxic compounds during the toxin transportation from the gut to the skin [28]. Therefore, such a strategy may enable frogs to cope with various exogenous chemicals and dramatically elevate skin secretion’s bioactivity by accumulating these compounds.

## 4. Materials and Methods

### 4.1. Animals, DRG Neurons and Skin Secretion Collection

Tree frogs (*Hyla japonica*, *n* = 55) used in this study were collected in Shangzhi, Heilongjiang province, China. The rats (*Rattus norvegicus*, *n* = 3) were purchased from Jiangsu province, China. As previously reported [29], a 3-V alternating current was used to stimulate the moistened skin manually, after which the secretion was washed using deionised water. For the preparation of the boiled secretion, the skin secretion underwent the water bath at 100 °C for 10 min. DRG neurons of rats were acutely dissociated and maintained in a short-term primary culture according to procedures as previously described [30]. All experiments involving animals conformed to the recommendations in the Guide for the Care and Use of Laboratory Animals of Northeast Forestry University. All experimental procedures were approved by the Institutional Animal Care and Use Committees at Northeast Forestry University (approval No: 2022070). All possible efforts were made to reduce the animals’ sample size and minimize their suffering.

### 4.2. PAX Purification and Identification

The lyophilized frog secretion was dissolved in methanol (2 mg/mL) and filtered by 0.22 μm MF-Millipore filters. The filtrate was separated and purified using C_18_ reverse-phase high-performance liquid chromatography (RP-HPLC; XBrige C_18_ column, 5 μm particle size, 4.6 × 250 mm Column). PAX was analyzed using a Q-Exactive Hybrid Quadrupole-Orbitrap mass spectrometer (Thermo Scientific, Waltham, MA, USA) coupled with a Dionex UltiMate 3000 UHPLC system (Thermo Scientific, Waltham, MA, USA) and identified through searching ChemSpider database.

### 4.3. Plasmids and Mutagenesis

The corresponding NCBI codes of cDNA sequences of KCNK18 orthologues are 445,371 (*Rattus norvegicus*) and 100,471,846 (*Ailuropoda melanoleuca*). The corresponding NCBI code of cDNA sequences of rat BK_Ca_ is 83,731 (*Rattus norvegicus*). These coding sequences were synthesized by Tsingke (Beijing, China) and subcloned into the pCDNA3.1 vector. All KCNK18 chimeras and single-point mutants were constructed using Fast Mutagenesis Kit V2 (SBS Genetech) following the manufacturer’s instructions. These channel chimeras and mutants were confirmed by DNA sequences.

### 4.4. Calcium Imaging

The dissociated rat DRG neurons were loaded with 3 μM Fluo-4 AM in Ringer’s solution (140 mM NaCl, 5 mM KCl, 2 mM MgCl_2_, 10 mM glucose, 2 mM CaCl_2_ and 10 mM HEPES, pH 7.4) for 40–60 min. After incubation, the cells were washed by 2 mL Ringer’s solution. The intact or boiled skin secretion (20 mg/mL) of tree frogs (Hyla japonica), 100 nM ChTx and 20 μM PAX were dissolved, respectively, in Ringer’s solution to excite DRG neurons. The changes in calcium fluorescence intensity of DRG neurons were acquired with an Olympus IX71 microscope with Hamamatsu R2 charge-coupled device camera controlled by the MetaFluor Software (Molecular Devices, San Jose, CA, USA). Fluo-4 was excited by a LED light source (X-Cite 120LED, Lumen Dynamics, Mississauga, ON, Canada) with a 500/20 nm excitation filter and a 535/30 nm emission filter.

### 4.5. Cell Culture and Transient Transfection

HEK293 cells were cultured in Dulbecco’s modified Eagle’s medium with 10% fetal bovine serum and 1% penicillin/streptomycin at 37 °C with 5% CO_2_. Cells were transiently transfected with DNA mixture (channel vectors and an enhanced green fluorescent protein (eGFP) using the Lipofectamine 2000 reagent (Invitrogen) following the instruction manual). Cells with fluorescence signals were selected for patch-clamp recordings 24 h after transfection. As expression of Nav1.8 is usually ineffective in HEK293 cells, rat Nav1.8 (3 µg) was transiently transfected into the neuroblastoma cell line N1E-115 by using a Nanofectin transfection kit (PAA Laboratories GMbH, Pasching, Australia). The culture condition of N1E-115 cells was the same as that of HEK 293 cells.

### 4.6. Electrophysiology

Whole-cell patches were recorded by using an EPC10 amplifier (HEKA) controlled by PatchMaster software (HEKA). Patch pipettes were made from borosilicate glass and fire-polished to a resistance of ~3 MΩ. For the potassium channel recording, the pipette solution contained 150 mM KCl, 3 mM MgCl_2_, 10 mM HEPES and 5 mM EDTA, pH 7.4, and the bathing solution contained 145 mM NaCl, 2.5 mM KCl, 3 mM MgCl_2_, 1 mM CaCl_2_, 10 HEPES, pH 7.4. The membrane potential was held at −80 mV, and the currents were elicited by a ramp voltage from −100 mV to +100 mV for 400 ms. For the TRP channel recording, both the pipette solution and the bathing solution contained 130 mM NaCl, 0.2 mM EDTA, 3 mM HEPES (pH 7.2). The membrane potential was held at 0 mV and the currents were elicited by two steps, 300 ms to 80 mV followed by 300 ms to −80 mV. For the sodium channel recording, the pipette solution contained 110 mM CsF, 15 mM NaCl, 1 mM CaCl_2_, 2 mM MgCl_2_, 10 mM TEA-Cl, 10 mM EGTA, 2 mM ATP-Mg and 10 mM HEPES (pH = 7.3). The bath solution contained 120 mM NaCl, 3 mM KCl, 2 mM MgCl_2_, 2 mM CaCl_2_, 10 mM HEPES, 30 mM glucose, 2 mM 4-aminopyridine (4-AP), 0.2 mM CdCl_2_ and 0.5 μM tetrodotoxin (TTX) (pH = 7.3). The membrane potential was held at −80 mV, and the currents were elicited by a steady voltage to −10 mV for 100 ms. The current signals were filtered at 2.9 kHz and sampled at 10 kHz. A gravity-driven system (RSC-200, Bio-Logic) was performed to perfuse bath or stimulated solutions. The patched cells were placed at the perfusion tube outlet. The solutions were flowed through separated tubes to minimize the mixing of the solutions.

### 4.7. Structural Model Construction

The structure of rat KCNK18 was modeled by alpha fold molecular modeling suite version v2.0 [31]. Rosetta Ligand application from Rosetta program suite version 2020.27 was used to dock paxilline to KCNK18 [32,33]. The structural model of KCNK18 was relaxed in a membrane environment using the RosettaMembrane application [34], and the model with the lowest energy scores was used as the input structure for docking. Based on the chimera and mutant experiments, paxilline was first placed into the pocket nearby F375 and then the paxilline was docked to the optimal location and progressed from low-resolution conformational sampling and scoring to complete atom optimization using all-atom energy function. The generated 10,000 models were first screened with a total energy score. The top 1000 models with the lowest total energy score were selected and further scored with the binding energy between paxilline and KCNK18. The top 10 models with the lowest binding energy were chosen as the final docking model.

### 4.8. Animal Behavior

An equivalent number of male and female rats were used, between 6 and 8 weeks old weighing between 200–250 g. Rats were maintained in wired cages under a 12 h light/dark cycle at 24 °C and provided with free access to laboratory-standard food and water. All studies were approved by the Animal Care and Use Committees at Northeast Forestry University and were consistent with the guidelines.

All tail intravenous injections were performed with a 0.3 × 13 mm needle. Physiological saline was used as the diluent and vehicle. Mice were injected with 10 mL/kg physiological saline, ChTx (20 mg/kg), or PAX (1, 10, or 20 mg/kg), respectively, (*n* = 4–8 for each group). After injection, each rat was placed in a holding cage for 10 min to recover before detecting the painful response. After that, the time of both eyes closing was recorded for 30 min to assess the pain in rats [22].

### 4.9. Statistical Analysis

Igor Pro (WaveMatrix, version 6.37) and Prism (GraphPad version 8.0.1) were used to analyze the experimental data from electrophysiological recordings. All values are given as mean ± SEM for the number of measurements indicated (n). Statistical significance was determined using the Student’s t-test and accepted at a level of *p* < 0.01. N.S. indicates no significance.

The currents of KCNK channel were measured at 100 mV, and normalized to the maximal currents in the absence of paxilline. The inhibition of PAX was calculated by the equation: Percent(inhibition) = (I_max_ − I_x_)/I_max_. I_X_ represents the KCNK18 current at 100 mV in the presence of concentration [x]. I_max_ represents the maximal current amplitude at 100 mV in the absence of paxilline.

EC_50_ values were calculated by fitting a Hill equation to the paxilline-induced dose-response relationship. Where n is an empirical Hill coefficient, EC_50_ is the concentration for the half-maximal effect of paxilline inhibition.

$$\frac{I_{x}}{I_{\max}}=1-\frac{{\left[X\right]}^{n}}{{\mathrm{EC}}_{50}^{n}+{\left[X\right]}^{n}}$$

τ_on_ and τ_off_ values were obtained from single exponential fits using the equations:

I_(t)_ = a_0_ + a_1_[1 − exp(−t/τ_on_)]

I_(t)_ = a_0_ + a_1_ exp(−t/τ_off_)

## Data Availability

All data generated or analysed during this study are included in this published article.
